# Periorbital Emphysema Due to Traumatic Pneumothorax

**DOI:** 10.7759/cureus.51691

**Published:** 2024-01-05

**Authors:** José Pedro Manata, Mariana Moniz Ramos, Tetiana Baiherych, Martim Alçada, João Matos Costa

**Affiliations:** 1 Internal Medicine, Hospital Distrital De Santarém, Santarém, PRT

**Keywords:** non-steroid anti-inflammatory drugs, pneumothorax, facial trauma, periorbital emphysema, crepitation, subcutaneous emphysema, allergic reaction, periorbital edema

## Abstract

Periorbital emphysema is rare and associated with facial trauma. Its main distinguishing feature is crepitation on palpation of the edema. It resolves spontaneously in a few days, but there are cases of orbital compartment syndrome that can lead to loss of vision.

Here we present the case of a 55-year-old male who came to the emergency department for bilateral periorbital edema associated with non-steroidal anti-inflammatory drug (NSAID) usage, for pain following a fall from a ladder. He was treated with antihistamines and corticosteroids, for presumed allergic reaction, but without response, and subsequently developed acute onset dyspnea. Chest x-ray revealed a left pneumothorax in the context of chest trauma. Chest CT scan after drain placement shows extensive subcutaneous emphysema. In the differential diagnosis of periorbital edema, in addition to allergic, inflammatory, and systemic causes, the traumatic ones should not be excluded.

## Introduction

Periorbital emphysema (PE) is a rare clinical manifestation, and a bilateral presentation is even less frequent [1,2]. This condition is due to the forced entry of air into the orbital or periorbital tissues and its retention by a unidirectional valve mechanism since the mucosa seals the entry point with increased intraorbital pressure [1,3,4]. The most common cause is face trauma, with fractures of the maxillary sinus, particularly the zygomatic arch and orbital floor [1-4]. 

PE has also been associated with surgical procedures (dental, ethmoidectomy, or rhinoplasty), barotrauma during diving, respiratory tract infections, and even procedures, such as upper gastrointestinal endoscopy, endoscopic retrograde cholangiopancreatography, and colonoscopy [3-6].

Clinically, it is characterized by edema of the orbital region, erythema, and local discomfort [7]. Crackling on palpation is the main clinical sign that differentiates this entity from others [2,4,5]. Normally, PE disappears spontaneously over a few days, as the air is reabsorbed [1,2,4]. However, it can progress and cause loss of vision, due to compartment syndrome of the orbit by compromising the optic nerve or retinal artery [1-4].

## Case presentation

**Figure 1:**
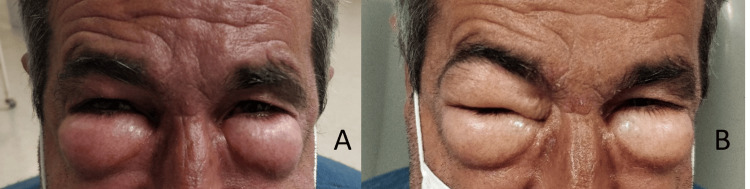
Bilateral periorbital swelling after taking NSAIDs for pain in the left rib cage. (A) On admission to the emergency department and (B) after 8 hours of evolution. NSAID: non-steroidal anti-inflammatory drug

A 55-year-old male came to the emergency department at the end of the day with bilateral periorbital edema, which he associated with taking a non-steroidal anti-inflammatory drug a few hours earlier for pain in the left chest wall after falling from a 2-meter high ladder (Figure 1, panel A).

His past medical history was significant for a recent diagnosis of hepatitis C secondary to prior intravenous drug use, a 40 pack/year smoking history, and terminal insomnia, medicated with olanzapine 10 mg at bedtime. On initial physical examination, he was calm, conscious, and with no signs of any breathing difficulties. Lung auscultation was normal, with bilateral vesicular murmur audible, adventitious noises were absent, and chest expansion was not impaired. Cardiac auscultation was rhythmic, with no audible murmurs or extra sounds. No signs of jugular engorgement. Vitals signs were as follows: blood pressure 163/104 mmHg, heart rate 104 beats per minute (bpm), peripheral oxygen saturation of 96% with room air, and no fever.

Given the clinical history, an allergic reaction to non-steroidal anti-inflammatory drugs (NSAIDs) was assumed, so he was treated with antihistamines and corticosteroids (clemastine 10 mg and hydrocortisone 200 mg intravenously). Laboratory tests, electrocardiogram (ECG), and x-ray of the left rib cage were performed. The laboratory study showed no significant alterations and the initial x-ray of the left chest wall only showed pulmonary contusion and rib fractures.

On clinical reassessment in the early hours of the following morning, he had worsening periorbital edema, with difficulty closing his eyelids. Despite everything, he remained without respiratory symptoms, with normal cardiac and pulmonary auscultation. Despite the stability shown at the time, it was decided to carry out an additional therapeutic test with adrenaline (0.5 mg intramuscularly). Given the lack of improvement, discharge was postponed and the patient was kept under observation for a few more hours. In the following hours, the edema continued to worsen, crepitation appeared, and a sudden onset of dyspnea (Figure 1, panel B).

**Figure 2:**
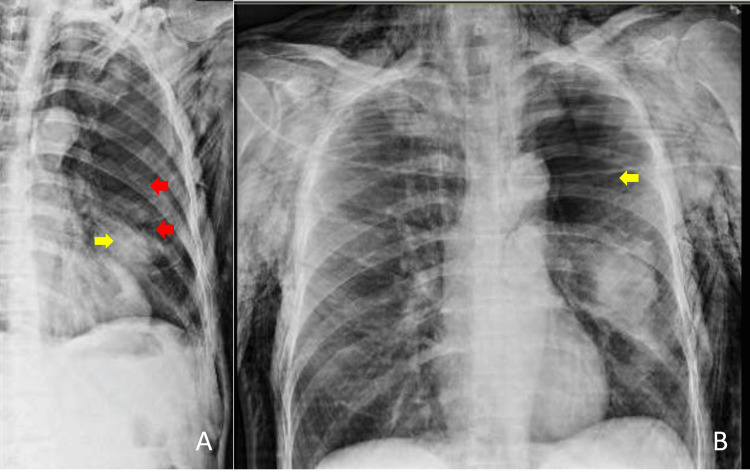
Urgent x-ray of the left rib cage and chest after sudden onset of dyspnea. (A) Pulmonary contusion (yellow arrow) and fractured ribs (red arrows), and (B) obvious subcutaneous emphysema and left pneumothorax (yellow arrow).

**Figure 3:**
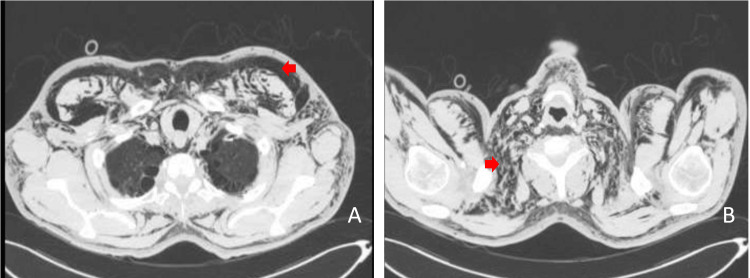
Extensive subcutaneous emphysema on chest computed tomography. Presence of air at (A) thoracic and (B) cervical levels (arrows).

A new left rib cage chest x-ray was carried out urgently (Figure 2, panels A and B), followed by a chest computed tomography (CT) scan which confirmed a left-sided pneumothorax with lung collapse and extensive subcutaneous emphysema (Figure 3, panels A and B).

A chest drain was placed, with a large amount of air coming out and relief of respiratory symptoms. The chest drain was removed on the sixth day of hospitalization, with periocular emphysema practically reabsorbed, and subcutaneous emphysema of the forearms and chest still palpable.

## Discussion

Most cases of PE due to pneumothorax are associated with bronchopleural fistulas, due to iatrogenic placement of a chest tube. Subcutaneous emphysema is more common from this procedure, but air infiltration into the soft tissues of the neck and face is a rare phenomenon [7-10].

Through a poorly understood fistulous route, the air invades the mediastinum and adjacent subcutaneous tissues, ascending through the vascular sheaths to the thoracic and cervical subcutaneous tissues, reaching the inferior orbital fissure, and culminating in emphysema [7,8].

By an identical mechanism, with a reverse circuit, after facial trauma the air can invade the neck spaces, creating subcutaneous emphysema with evolution to pneumomediastinum and pneumothorax [8]. PE is clinically classified into four stages as follows: stage I - no clinical signs, only radiological; stage II - dystopia/proptosis of the globe; stage III - loss of vision due to compression of the optic nerve; and stage IV - occlusion of the central retinal artery [11-14].

The case described in this study was initially based on the assumption of an allergic reaction to NSAID, but given the lack of response to the therapy instituted with antihistamines and corticosteroids, and after a better assessment of the clinical history, it was confirmed that this hypothesis was unlikely, given that the patient had already taken other doses of NSAIDs without any associated secondary reaction.

In the differential diagnosis of periorbital edema, in addition to allergic, inflammatory, and systemic causes, the traumatic ones and iatrogenesis associated with invasive procedures should not be excluded [8-12]. In this case, there was no evidence of spontaneous bronchopleural fistulas or iatrogenic placement of the chest tube, since it was placed after the PE had already formed [7,10,12].

This seems to us to be a case of PE after pneumothorax in the context of thoracic trauma with total spontaneous resorption after placement of a chest drain. According to Hunts et al. classification of orbital emphysema, this patient presented in stage 1, without dystopia or proptosis of the globe or loss of vision. As such, ophthalmology consultation was not required in this case [14,15]. Despite the already known mechanism by which air invades and ascends to the cervical subcutaneous tissues, passing through the inferior orbital fissure, its entry via other dehiscences of the facial bones cannot be ruled out.

It's worth remembering the particularity of this case, given the history of past drug use. Chronic intranasal cocaine use results in repeated vasoconstriction of the nasal mucosa, with ischemia and subsequent mucosal necrosis. Repeated exposure is followed by damage to cartilage and bone structures, in particular the structural support of the midline of the face [16-18]. This cocaine-related destructive process is commonly known as "cocaine-induced midline destructive lesion" (CIMDL) [16,17].

In this particular clinical case, although there is no knowledge of direct trauma to the face, the previous exposure to cocaine may have caused some fragility in these bone structures, and thus led to the early appearance of PE, without any other clinical expression of subcutaneous emphysema.

## Conclusions

We describe a rare case of PE due to traumatic pneumothorax, with no known trauma to the face. In the differential diagnosis of periorbital edema, the appearance of the sign of crepitation on palpation of the edema led to the diagnosis of subcutaneous emphysema after chest trauma.

A more precise clinical history made it possible to rule out allergic causes and leaves open for discussion the possibility of fragility of facial bone structures in the context of previous cocaine consumption, as well as alerting us to monitor the patient more closely given the potential for other pathologies associated with CIMDL. The rapid diagnosis and placement of the chest tube halted the progression of the PE to the most advanced stages.
